# Vaccinia virus tiantan strain is inefficient in eliciting cross-reactive immunity against the emerging monkeypox virus strain

**DOI:** 10.1080/22221751.2024.2306967

**Published:** 2024-01-19

**Authors:** Lingqian Tian, Yongli Zhang, Qiuhong Liu, Lianguo Ruan, Fuli Ren, Yang Han, Yanfang Zhang, Lei Yang, Sha Li, Hao Sun, Yecheng Zhang, Yuan Zhou, Rongjuan Pei, Fei Deng, Chaolin Huang, Xinwen Chen, Yun Wang

**Affiliations:** a State Key Laboratory of Virology, Center for Biosafety Mega-Science, Wuhan Institute of Virology, Chinese Academy of Sciences, Wuhan, People’s Republic of China; b University of Chinese Academy of Sciences, Beijing, People’s Republic of China; c Department of Infectious Diseases, Wuhan Jinyintan Hospital, Tongji Medical College of Huazhong University of Science and Technology, Wuhan, People’s Republic of China; d Center for Translational Medicine, Wuhan Jinyintan Hospital, Tongji Medical College of Huazhong University of Science and Technology, Wuhan, People’s Republic of China; e Guangzhou Institute of Pediatrics, Guangzhou Women and Children's Medical Centre, Guangzhou Medical University, Guangzhou, People’s Republic of China; f Tongji Medical College of Huazhong University of Science and Technology, Wuhan Jinyintan Hospital, Wuhan, People’s Republic of China; g Guangzhou National Laboratory, State Key Laboratory of Respiratory Disease, Guangzhou Medical University, Guangzhou, People’s Republic of China

Dear Editor,

Orthopoxviruses (OPXVs) are a genus of large DNA virus that are responsible for some of the most virulent diseases of humans and animals, including variola virus (VARV), which causes smallpox, and monkeypox virus (MPXV), which causes mpox. Both VARV and MPXV were classified as category one human infectious pathogen by the Ministry of Health of the People’s Republic of China in 2006. Beginning in early May 2022, an unexpected outbreak of mpox, caused by an emerging MPXV strain (Clade IIb) [1], has surfaced and is currently ongoing in over 116 countries around the world (https://worldhealthorg.shinyapps.io/mpx_global/). In the mainland China, the first imported case of mpox was reported in Sept, 2022, and at least 491 mpox cases were confirmed within one month of July, 2023 (https://www.chinacdc.cn/jkzt/crb/qt/szkb_13037/).

Since OPXV members shared high sequence similarities, a less virulent member of OPXV, vaccinia virus (VACV), was employed as a smallpox vaccine in the Smallpox Eradication Programme (SEP) led by the World Health Organization from 1966 to 1980 (https://www.who.int/health-topics/smallpox). In China, the VACV Tiantan strain (VTT) is the traditional smallpox vaccine and was routinely inoculated in this country before it was discontinued in 1981. Cross-neutralization antibody (cNAb) induced by VACV has been shown to play an essential role in protection against pathogenic OPXVs including VARV and MPXV [2]. To control the current wave of mpox outbreak in China, an urgent task is to determine whether VTT is efficient in eliciting cNAb against MPXV. A recent study showed that MPXV-cNAb could be detected in as low as 26.7% of individuals who had been vaccinated with VTT before 1981 [3]. However, it remains undetermined whether the low level or absence of MPXV-cNAb is caused by the inefficiency of VTT to induce MPXV-cNAb, or if the neutralization titer wanes over a course of four decades [4]. The answer to this question is of particular importance for the government and vaccine companies to decide whether or not to restart the production line of VTT to control the current mpox outbreak in China.

To address this important question, we immunized mice with VTT and investigated whether the induced humoral and cellular immunities can cross-react with the emerging MPXV strain (Clade IIb) isolated in 2023 in Wuhan, China (IVCAS 6.9141). The experimental design is diagrammed in Figure 1A. Six-week-old female BALB/c mice (*n* = 6) were intraperitoneally vaccinated with VTT of 1 × 10^4^, 1 × 10^5^, and 1 × 10^6^ plaque forming units (PFUs). At 21 days after the prime vaccination, mice received a boost inoculation. Mice were euthanized at day 35 following the prime vaccination and their sera and splenocytes were collected to analyze the humoral and cellular immune responses.

**Figure 1.**
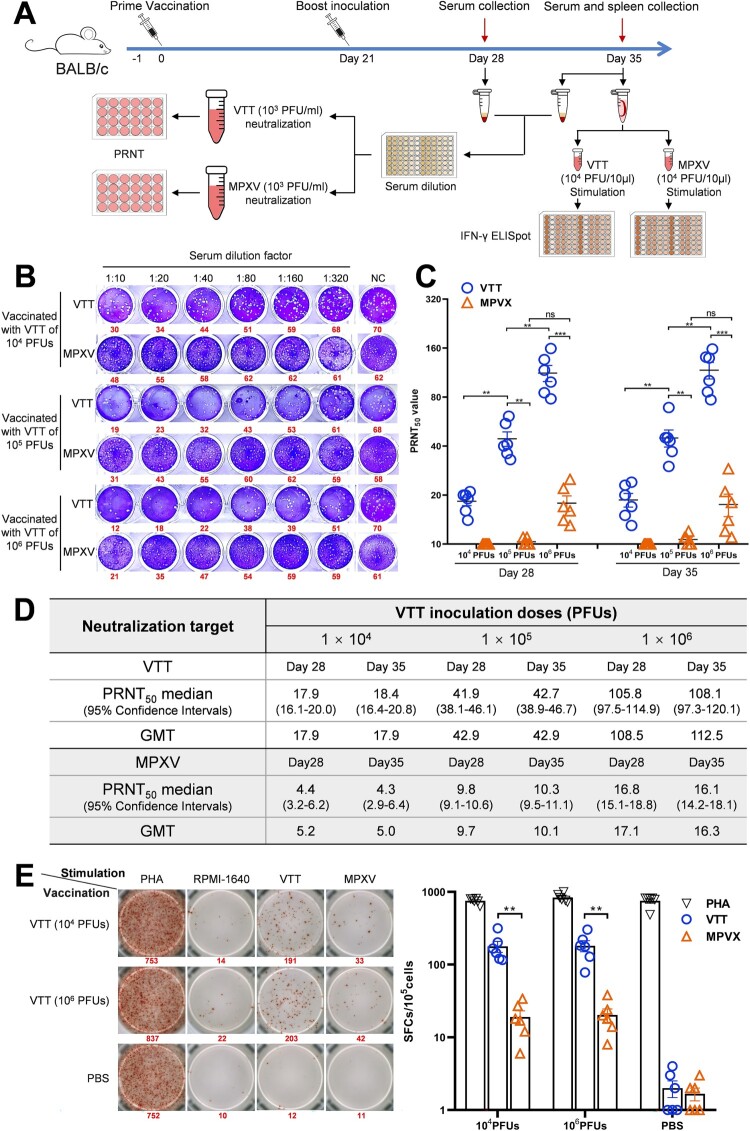
VTT is inefficient in induction of cross-reactive immunity against MPXV in mice. (A). Diagram of the experiment. Mice were immunized with VTT at different doses ranging from 1 × 10^4^ to 1 × 10^6^ PFUs, and the sera and splenocytes were collected at indicated days post the boost vaccination. Serially diluted sera were then mixed with live VTT and MPXV before being incubated with fresh Vero E6 cells. A standard PRNT was performed to evaluate the NAb against VTT and cNAb against MPXV. Splenocytes were exposed to heat-inactivated VTT and MPXV, respectively. An ELISpot assay was then performed to quantify IFN-γ secreting cells upon viral stimulation. (B). Viral plaques induced by VTT and MPXV. The plaques were stained with crystal violate and were quantified visually. The serum dilution factors, VTT inoculation doses, and the median numbers of plaques (bold fonts in red) were designated in line. (C). Neutralizing activity of sera against VTT and MPXV was determined and shown as PRNT_50_. The lines (in black) indicate the geometric mean and the whiskers (in blue) indicate the standard error of mean. (D). PRNT_50_ medians and GMTs for VTT and MPXV neutralization. (E). Cellular immune response upon viral stimulation. Splenocytes extracted from PBS- or VTT-vaccinated mice were stimulated with PBS, phytohemagglutinin (PHA, 10 mg/ml), and heat-inactivated VTT and MPXV (10^4^ PFU/10 μl). The number of IFN-γ secreting cells was visualized and counted using an AID MultiSpot Reader System MSR07. The median numbers of spots (bold fonts in red) were designated in line (left panel). Statistical analysis was performed using the one-way ANOVA tests and *t*-test analysis (right panel). *P* values <0.05 were considered statistically significant (ns, not significant, *p < 0.05, **p < 0.01, ***p < 0.001).

To investigate the humoral immune response, fifty PFUs of VTT or MPXV were mixed 1:1 (V/V) with serially diluted serum samples (1:10, 1:20, 1:40, 1:80, 1:160, 1:320), respectively. After a one-hour incubation, each serum-virus mixture was added to confluent Vero E6 cells plated on 24-well plates. A plaque reduction neutralization test (PRNT) was performed to compare the neutralization activity of the serum against VTT and MPXV (Figure 1B). NAb or cNAb titers led to a 50% reduction in plaque numbers (PRNT_50_) and the corresponding geometric mean titer (GMT) were calculated.

At day 28 after the prime vaccination, in the sera of mice inoculated with 1 × 10^4^ PFUs of VTT, the GMT of NAb against VTT was 17.9, whereas cNAb against MPXV can hardly be detected. When the inoculated dose was increased to 1 × 10^5^ PFUs, the GMT of MPXV-cNAb could be detected as low as 9.7, whereas the GMT of VTT-NAb was lifted to 42.9. This result suggested that at least 10-fold higher VTT inoculation dose is required to elicit detectable MPXV-cNAb, as compared to the dose needed to elicit VTT-NAb. Immunizing the mice with 1 × 10^6^ PFUs resulted in an increase of the MPXV-cNAb GMT to 17.1, yet it was still 6.3 times less than 108.5 of VTT-NAb GMT. (Figure 1C,D). Notably, there was no significant increase in the viral neutralization titer of sera at day 35 after the prime vaccination. (Figure 1C,D).

Such a reduced efficiency in eliciting MPXV-cNAb by VTT vaccination is not unexpected, as we previously demonstrated that certain immuno-dominant protective antigens of MPXV react poorly with VTT-induced antibodies [5]. Given the effectiveness of VTT and other VACV strains in eliminating smallpox, it should be applicable to use VTT as an efficient mpox vaccine if it can induce cNAb against MPXV as effectively as against VARV. However, due to the lack of VARV, we cannot perform a PRNT to evaluate the neutralization activity of the VTT-induced antibodies against VARV. Dryvax is another smallpox vaccine widely-used in the SEP from 1966 to 1980. Similar to VTT, Dryvax is also derived from VACV. Therefore, we believe it is feasible to see Dryvax as a surrogate of VTT in terms of inducing cNAb against VARV. A previous study demonstrated that the GMT of Dryvax-induced cNAb against VARV displayed a 1.3-fold reduction from the GMT of NAb against Dryvax itself [6]. Compared to the slight decrease in the efficiency of inducing VARV-cNAb by Dryvax, there was a considerable loss (>6-fold reduction) in the efficiency of eliciting MPXV-cNAb by VTT. The result suggested that VACV was more suitable to serve as a smallpox vaccine rather than an mpox vaccine. As supportive evidence, the FDA-approved mpox vaccine MVA-BN, a replication-deficient VACV can induce detectable MPXV-cNAb in only 63% recipients after two shots [7].

Although humoral immune response is generally believed to play an essential role in preventing OPXVs infection [2], we also detected the cellular immune response induced by VTT. The murine splenocytes were stimulated with heat-inactivated VTT or MPXV (10^4^ PFU/well) and the IFN-γ ELISpot assay was performed [8]. The median number of IFN-γ secreting cells in mice vaccinated with 1 × 10^4^ PFUs VTT reached 177 spot-forming cells (SFCs)/10^5^ cells after exposure to VTT, but decreased to 19 SFCs/10^5^ cells upon MPXV stimulation (Figure 1E). The 9.3-fold reduction suggested that VTT is also inefficient in eliciting cross-reactive cellular immunity against MPXV. Interestingly, the highest VTT vaccination dose (1 × 10^6^ PFUs) only slightly increased IFN-γ secreting cells to 181 SFCs/10^5^ cells upon VTT stimulation, or 20 SFCs/10^5^ cells after exposure to MPXV (Figure 1E).

In China, more than 96% mpox patients were men who have sex with men (MSM) (https://www.chinacdc.cn/jkzt/crb/qt/szkb_13037/), and a considerable portion of MSM (ranging from 2.5% to 12.1%) were living with human immunodeficiency virus (HIV) [9]. Owing to the risk of systemic VACV infection, it is not advisable to vaccinate people living with HIV using replication-competent VACV including VTT. Therefore, the inefficiency of VTT in eliciting cross-reactive immunity against the emerging MPXV strain, along with safety concerns in MSM, has highlighted the pressing need for developing improved vaccines to combat the emerging mpox.

## References

[1] Happi C, Adetifa I, Mbala P, et al. Urgent need for a non-discriminatory and non-stigmatizing nomenclature for monkeypox virus. PLoS Biol. 2022 Aug;20(8):e3001769. doi:10.1371/journal.pbio.3001769 35998195 PMC9451062

[2] Edghill-Smith Y, Golding H, Manischewitz J, et al. Smallpox vaccine-induced antibodies are necessary and sufficient for protection against monkeypox virus. Nat Med. 2005 Jul;11(7):740–747. doi:10.1038/nm1261 15951823

[3] Zeng Y, Liu X, Li Y, et al. The assessment on cross immunity with smallpox virus and antiviral drug sensitivity of the isolated mpox virus strain WIBP-MPXV-001 in China. Emerg Microbes Infect. 2023 Dec;12(1):2208682. doi:10.1080/22221751.2023.2208682 37128898 PMC10177700

[4] Li E, Guo X, Hong D, et al. Duration of humoral immunity from smallpox vaccination and its cross-reaction with Mpox virus. Signal Transduct Target Ther. 2023 Sep 15;8(1):350. doi:10.1038/s41392-023-01574-6 37709783 PMC10502045

[5] Yang L, Chen Y, Li S, et al. Immunization of mice with vaccinia virus Tiantan strain yields antibodies cross-reactive with protective antigens of monkeypox virus. Virol Sin. 2023 Feb;38(1):162–164. doi:10.1016/j.virs.2022.10.004 36272712 PMC9580254

[6] Hughes CM, Newman FK, Davidson WB, et al. Analysis of Variola and Vaccinia Virus Neutralization Assays for Smallpox Vaccines. Clin Vaccine Immunol. 2012 Jul;19(7):1116–1118. doi:10.1128/CVI.00056-12 22593237 PMC3393368

[7] Zaeck LM, Lamers MM, Verstrepen BE, et al. Low levels of monkeypox virus-neutralizing antibodies after MVA-BN vaccination in healthy individuals. Nat Med. 2023 Jan;29(1):270. doi:10.1038/s41591-022-02090-w 36257333 PMC9873555

[8] Wang Y, Kan S, Du S, et al. Characterization of an attenuated TE3L-deficient vaccinia virus Tian Tan strain. Antiviral Res. 2012 Dec;96(3):324–332. doi:10.1016/j.antiviral.2012.10.002 23084929

[9] Choi KH, Liu H, Guo Y, et al. Emerging HIV-1 epidemic in China in men who have sex with men. Lancet. 2003 Jun 21;361(9375):2125–2126. doi:10.1016/S0140-6736(03)13690-2 12826438

